# Editorial: Diabetes surveillance in Germany – status and perspectives

**DOI:** 10.25646/5985

**Published:** 2019-06-27

**Authors:** Christa Scheidt-Nave, Andrea Icks

**Affiliations:** 1 Robert Koch Institute, Berlin; 2 Institute for Health Services Research and Health Economics, German Diabetes Center (DDZ), Leibniz Institute for Diabetes Research at Heinrich Heine University Düsseldorf; 3 Institute for Health Services Research and Health Economics, Faculty of Medicine, Heinrich Heine University Düsseldorf

Against the backdrop of the great public health importance of diabetes mellitus, receiving funds from the Federal Ministry of Health (BMG), the Robert Koch Institute (RKI) has begun a research project to establish diabetes surveillance in Germany. In the public health context, surveillance refers to a systematic long-term collection and analysis of health data to facilitate decisions regarding the planning, implementation and evaluation of public health measures [1]. Diabetes surveillance is thereby fundamentally concerned with transparently providing key information on diabetes in Germany for actors in health policy, health research and practice as well as the general public. This includes information on risk factors, disease rates, disease outcomes and the quality of medical care. Within the first four-year project phase (2015-2019), a scientific framework concept with four fields of action and 40 key indicators was developed, data sources to map these indicators were established, and formats for user-oriented reporting created. An interdisciplinary scientific advisory board has continuously guided the project [1].

Type 2 diabetes is the dominant form of diabetes at adult age and as one of the globally most common chronic diseases now stands in the focus of international action plans for the prevention of noncommunicable diseases [2]. Elevated blood sugar levels as a result of decreased insulin action (insulin resistance) are the hallmark of this type of diabetes. Besides genetic factors, the important risk factors for type 2 diabetes and other common noncommunicable diseases include in particular older age and health risks such as obesity, physical inactivity and smoking, which have a high prevention potential. As behaviour-related risk factors are closely settings bound, i.e. tied to people's social, cultural and work environments or their physical environments, this translates into a responsibility for the whole of society to promote the prevention and containment of type 2 diabetes, other frequent noncommunicable diseases and contribute to reducing health inequalities [3, 4]. Next to type 2 diabetes, which is the most frequent form of diabetes at adult age, diabetes surveillance also covers the far rarer type 1 diabetes that usually develops at child and adolescent age as well as gestational diabetes. Both primary data from the RKI from national health surveys as well as disease registry data and disease management program (DMP) data for diabetes types 1 and 2 as well as routine billing data from the statutary health insurance system for secondary use (called secondary data) are continuously being used to fill indicators in the four fields of action: 1. Reducing diabetes risks, 2. Improving early detection and treatment of diabetes, 3. Limiting diabetes complications, 4. Reducing the disease burden and costs. To ensure the use of external data sources in the long term, develop potential uses, identify and reduce data use limits, annual tenders for cooperation projects have been published in the context of diabetes surveillance and selected based on standardised criteria. The articles in this issue of the Journal of Health Monitoring on New Results from Diabetes Surveillance in Germany provide an overview of the key results diabetes surveillance has provided towards the end of the first project phase set to conclude at the end of 2019.

In the first article, Heidemann et al. ask whether diabetes-related social inequalities have increased for adults in Germany over time. The findings are based on data from continuous health monitoring at the RKI with information that was collected in national population representative interview and examination surveys on adult health in Germany between 1997 and 1999 (German National Health Interview and Examination Survey, GNHIES98) and 2008 to 2011 (German Health Interview and Examination Survey for Adults, DEGS1). As the results convincingly show, levels of education have a clear impact on diabetes prevalence and estimated 5-year diabetes risks in Germany, however, not on quality of life and certain aspects of quality of care. During both survey points and for both genders, diabetes prevalence is considerably higher among adults with low education compared to those with high education. This finding applies for both medically diagnosed diabetes as well as to unknown diabetes. That the prevalence of unknown diabetes has dropped across all education groups, with a concomitant increase in medically diagnosed diabetes and relatively constant overall prevalence could indicate improved early detection. It is, however, problematic that over the same period the education gap in the estimated 5-year diabetes risk among adults in Germany has continued to widen. For both genders, a significant decline can only be seen in the high education group. This represents a major challenge for health promotion and primary care, the successes of which should be closely monitored at the population level. Regarding the developments of the impact of diverging levels of education on quality of care, the results do not indicate pronounced levels of educational inequality at either survey point. Over time, clear improvements in particular in the low education group are visible. This applies to meeting guideline-based objectives for glycated haemoglobin (HbA1c or long-term blood sugar), blood pressure and blood lipids, self-measurement of blood sugar, and regularly attending medical examinations of the ocular fundus and the feet. At least between 1997 and 1999, taking statins was less common in both genders in the low education group in comparison to the medium or high education groups. However, these differences may not be statistically significant due to the very low number of statin users at the time. Yet, as even countries with universal access to their health care system report social differences in the quality of care [5-7], there is a need for further research here. In future, it will be important to include hard endpoints such as overall mortality and cardiovascular complications next to figures on care processes and target achievement criteria for the regulation of blood sugar, blood pressure and cholesterol values. In addition, the operationalisation of social inequality should be extended beyond merely education. Observations from Germany show that in children and adolescents with type 1 diabetes both lower individual social status and a higher regional deprivation index are associated with poorer quality of care [8-10].

The second article by Rosenbauer et al. shows how the data gaps on type 1 diabetes and type 2 diabetes in children and adolescents in Germany could be closed in the future. In contrast to the civilisation disease type 2 diabetes, type 1 diabetes, the second major form of diabetes mellitus, is much less common and has often already developed in childhood or adolescence. Type 1 diabetes is caused by a not hitherto fully understood autoimmune reaction that destroys the pancreas’ insulin-producing cells. The consequence is a lifelong insulin dependency that places high demands on the self-management of patients and the quality of medical care. Not least, this applies with regard to the important technological advances made in insulin pumps and measuring instruments for continuous glucose measurement in the subcutaneous tissue as well as on-demand insulin delivery by means of so-called ‘closed loop systems’ [11, 12]. Estimates on the prevalence of type 1 diabetes among children and adolescents in Germany have been based on (one national and three regional) incidence registries since the mid-1990s with a high degree of completeness [13, 14]. Still lacking in Germany are standardised comparable estimates over time on the prevalence and incidence of type 1 diabetes among adults and overall estimates across all age groups. An increase in type 2 diabetes in children and adolescents is generally suspected, yet so far no time series based on systematic and continuous data collection has been established. Both the regional registry in North Rhine-Westphalia (NRW) and the national DPV registry (Diabetes-Patienten-Verlaufsdokumentation) meanwhile include all newly diagnosed cases of type 1 diabetes in adults and type 2 diabetes in children and adolescents - however coverage is not complete. Based on statistical methods (capture-recapture), data from the registry in NRW was used to estimate coverage rates. Assuming varying degrees of completeness, capture-adjusted estimates of the prevalence and incidence of type 1 diabetes in adults aged over 18 were established based on German DPV registry data. For type 1 diabetes in adults aged over 18, current prevalence estimates (493 per 100,000 people or a total of 341,000 people for the year 2016) and incidence (6.1 per 100,000 person years or 4,150 new patients per year) are significantly higher than the earlier estimates based on analyses of statutory health insurance data. In the synopsis with the data on children and adolescents up to 18 years already available, the results for Germany indicate that there are currently 373,000 people with type 1 diabetes, implying an estimated absolute number of 7,265 new patients per year. For type 2 diabetes among children and adolescents, in addition to estimates based on the available NRW and DPV registry data, further surveys were conducted in diabetes registry clinics in Baden-Württemberg and Saxony. The results for the period 2014-2016 show that type 2 diabetes among children and adolescents in Germany remains a rare disease with a prevalence of an estimated 12 to 18 per 100,000 persons, a total of about 950 children and adolescents and an estimated 175 new cases per year. Based on the rare occurrence of type 1 diabetes and especially of type 2 diabetes in children and adolescents, population-based samples are poorly suited to record disease rates over time. Analyses of routine data, too, from statutory health insurances have their limits because of often unclear or incomplete coding and changing case definitions. An expansion of the available registry data with improved completeness detection is therefore essential for reliable, comparable trend estimates over time on the disease burden and need for care in the context of diabetes surveillance.

The third article by Schmidt et al. discusses the question of secondary data. Secondary data refers to data that has been primarily collected for a different purpose [15]. This can be data which has been collected for a study and is then analysed to answer a new research question. Often, secondary data is data that is primarily collected for routine or billing purposes, for example the data of social security institutions such as health or pension insurances, medical record data or disease management program data. It has clear disadvantages compared to primary data such as the survey data referred to above. For example, it does not usually contain any patient-reported variables (such as quality of life). The validity of this data for the research question needs to be carefully evaluated. However, such data also has a number of relevant advantages. Large populations can be observed. There is no selection due to non-participation. Regional stratifications are possible. Some events, which are of key importance as outcome indicators, are well and fully described, for example amputations or strokes. Furthermore, process variables can be collected that cannot be validated easily in interviews, such as the number of doctors’ appointments or certain examinations, that patients either do not reliably remember or are not aware of. In addition, longer periods of time can be surveyed in a tight temporal sequence [15]. Many key findings on diabetes in Germany are based on secondary data, such as diabetes prevalence, stroke and amputation incidence [16, 17], referrals to doctors [18], and cost of medical care [19]. However, little data has routinely been available so far and separate studies are required. In addition, methodological aspects need to be critically reflected, such as differences between different health insurance organisations regarding the composition of the insured population or the suitability of routine data to assess health events [20]. Using routine data in diabetes surveillance therefore depends on the one hand on establishing the validity of data sources and possibly reviewing the methodology. On the other hand, it is important to determine the availability and potential of continuity in terms of a regular provision of data for surveillance. Such an approach is highly important because 14 of the 40 indicators of diabetes surveillance exclusively use secondary data and a further eleven indicators, which are mainly based on RKI health monitoring data, in addition require secondary data. The article describes the two ‘work packages’ diabetes surveillance has devoted to secondary data. First, cooperation projects were conducted in which external partners examined data sources regarding their validity, availability and usability for surveillance. Secondly, based on the DaTraV dataset (according to the Data Transparency Regulations), criteria for the operationalisation of diabetes prevalence were defined and the data evaluated.

The following projects were realised:

1. On the basis of data from the diagnosis related groups statistics (DRG statistics), trends in outpatient-sensitive hospital cases in diabetes mellitus were analysed: There was a marked decrease in age-adjusted amputation rates, which - at least partially - could be related to improvements in care [21].
2. The usability of DMP data for diabetes surveillance was tested: It turned out that DMP data, despite some limitations (selection, questionable validity of the documentation), can provide important results, such as the achievement of quality objectives and the implementation of care in accordance with guidelines.
3. The presentation of relevant quality of care indicators based on AOK data was analysed: The project highlighted that adequately prepared secondary data has the potential to close data gaps in diabetes surveillance. Based on the project results, four further indicators were included in the diabetes surveillance indicator set.
4. DaTraV data was used to project future scenarios of diabetes development in Germany: Assuming that demographic developments continue and diabetes prevalence remains constant, the absolute number of persons with diagnosed type 2 diabetes would increase by 21% between 2015 and 2040 [22].
5. The potential value of geocoding services data to make statements on the obesogenicity of an environment, which means an environment that potentially increases obesity, was analysed: The project developed a method that allows areas with obesogenic and/or protective environmental factors to be identified. Potentially, this could be used in diabetes surveillance.
6. Healthy life years and life years lost were calculated as indicators for diabetes burden: The majority of models indicated an increase in healthy life years between 2015 and 2040 as well as a relative decline in lost life years by up to 64%.
7. The incidence trends for renal replacement therapy were analysed based on medical records and a concept was established for analysis based on the data of statutory health insurances. Furthermore, the usability of DaTraV data for monitoring the trends in terminal renal insufficiency was analysed: Unlike for other complications, no decline in the incidence of renal replacement therapy was observed during the 2000s. Currently, the trend between 2002 and 2016 is being analysed. An analysis of DaTraV data showed that validation by comparing data on renal replacement therapy with data on the diagnosis of terminal renal replacement therapy can provide insights as to whether information on a terminal renal replacement therapy is continuously available for diabetes surveillance based on DaTraV data and can therefore provide a valid measure.

The second work package, in cooperation with experts from epidemiology and health services research, developed a reference definition for the future presentation of the documented prevalence of diabetes in the context of diabetes surveillance, which is based on DaTraV data. Overall, the article clearly shows that secondary data is a key element to map indicators of diabetes surveillance that substantially complements RKI health monitoring. This data can provide time series for the development of numerous indicators.

In the final article, Reitzle et al. consider the central question of data processing and dissemination of the results of surveillance to actors in politics, research and practice. Taking diabetes as a concrete example, between April and September 2018, the models and experiences in 46 countries, among them 28 EU member states, five further European nations and 13 non-European OECD member states, were taken stock of. Structured online interviews with public health and health policy experts in 27 countries as well as structured internet searches (key word searches on the websites of public health institutes, health ministries, statistical offices and keyword-based searches on Google) provided the basis. There were 19 countries in which no interviews could take place. Information from web searches that were not available either in English, French or German were translated into English and included where appropriate. The results highlight the great importance awarded to health reporting on diabetes in 80% of the 46 countries (n=37), which is underwritten in more than three quarters of these countries by a national diabetes strategy or a national diabetes action plan. Reporting is mainly based on indicators (n=29) and in many countries (n=21) not diabetes specific but set within the context of an indicator-based reporting on a number of noncommunicable diseases. As in Germany, many countries use a diverse set of data sources in reporting, in many cases primary data from health surveys, but they also use secondary data from available routine data. The results of this analysis are already being considered in the development of formats for continuous diabetes reporting in Germany. The first report (print and online) will be published at the end of the first project phase of diabetes surveillance at the end of 2019. During the second project phase (2020-2021), expanding cooperation with actors in politics, public health research and practice in Germany, as well as with international public health institutes, will play a fundamental role.
